# The flexibility and dynamics of the tubules in the endoplasmic reticulum

**DOI:** 10.1038/s41598-017-16570-4

**Published:** 2017-11-28

**Authors:** Pantelis Georgiades, Victoria J. Allan, Graham D. Wright, Philip G. Woodman, Parinya Udommai, Manloeng A. Chung, Thomas A. Waigh

**Affiliations:** 1 Biological Physics, School of Physics and Astronomy, The University of Manchester, Manchester, M13 9PL UK; 2 Faculty of Biology, Medicine and Health, Michael Smith Building, The University of Manchester, Manchester, M13 9PT UK; 3 Photon Science Institute, Alan Turing Building, The University of Manchester, Oxford Rd., Manchester, M13 9PL UK; 4 IMB Microscopy Unit, Institute of Medical Biology, A*STAR, 8A Biomedical Grove, #06-06 Immunos, Singapore, 138648 Republic of Singapore

## Abstract

The endoplasmic reticulum (ER) is a single organelle in eukaryotic cells that extends throughout the cell and is involved in a large number of cellular functions. Using a combination of fixed and live cells (human MRC5 lung cells) in diffraction limited and super-resolved fluorescence microscopy (STORM) experiments, we determined that the average persistence length of the ER tubules was 3.03 ± 0.24 μm. Removing the branched network junctions from the analysis caused a slight increase in the average persistence length to 4.71 ± 0.14 μm, and provides the tubule’s persistence length with a moderate length scale dependence. The average radius of the tubules was 44.1 ± 3.2 nm. The bending rigidity of the ER tubule membranes was found to be 10.9 ± 1.2 *kT* (17.0 ± 1.3 *kT* without branch points). We investigated the dynamic behaviour of ER tubules in live cells, and found that the ER tubules behaved like semi-flexible fibres under tension. The majority of the ER tubules experienced equilibrium transverse fluctuations under tension, whereas a minority number of them had active super-diffusive motions driven by motor proteins. Cells thus actively modulate the dynamics of the ER in a well-defined manner, which is expected in turn to impact on its many functions.

## Introduction

The endoplasmic reticulum (ER) is a remarkably complex organelle found in eukaryotic cells, enclosed in a continuous membrane, which spans the entirety of the cell. Two distinct morphological regions can be distinguished in the ER; one that surrounds the nucleus and appears flatter and sheet-like, and one that spans the peripheral regions of the cell, which contains a network of tubules^1,2^, with occasional lamellar patches^3^. Furthermore, the ER network is not static in time; it is highly dynamic, continuously undergoing rearrangements, tubule formations, extensions and movements^4–6^. As the largest organelle in eukaryotic cells, the ER has been shown to be involved in many of the cell’s functions, such as protein synthesis, quality control and degradation^7^, ion and lipid exchange^8^, the promotion of endosomal translocation^9^ and apoptotic regulation^10^.

Due to recent advances in imaging techniques and the resultant access to higher resolution images, new insights into the structure of what were considered to be flat ER sheets have been obtained^2,3^. Using a combination of live and fixed cell super-resolution fluorescence imaging methods, Nixon-Abell *et al*. were able to identify ER tubules in regions that were previously believed to be ER sheets, by investigating gaps in the sheet-like peripheral ER^11^. These were previously unresolved due to spatial and temporal resolution limitations of the imaging techniques, and their existence was confirmed using electron microscopy. This group has also performed studies into the dynamic behaviour of the ER, by tracking three-way junctions between the tubules, which highlighted the dynamic oscillations of ER tubules and junctions, as well as its highly variable morphology^11,12^.

The polymer physics of semi-flexible fibres is now a rich and well developed field of research^13,14^. Standard calculations using the equipartition theorem allow the persistence length (*L*_*p*_) of semi-flexible fibres to be related to the elasticity of the fibres. The persistence length (*L*_*p*_) is defined as the length scale over which angular correlations in the tangent direction along the backbone decorrelate as a function of the distance along the backbone contour. The persistence length is a key basic property of polymers and it helps to quantify their rigidity. In practice, a polymer chain will show *flexible* behaviour if its length exceeds its persistence length, whereas it will behave as a *rigid rod* at lengths much smaller that its persistence length and a *semi-flexible* chain on length scales on the order of its persistence length^15–17^. In addition to the end-to-end distance and cross-sectional diameter, the persistence length is one of the most important quantities that describe the conformation of a fibre. Many of the cytoskeletal proteins inside cells, such as actin (*L*_*p*_ ~ 10 ± 1 μm)^18^ and microtubules (*L*_*p*_ ~ 5200 μm)^15^, have been studied in detail to understand their contributions to the mechanics of cells, such as estimating the energy stored during the bending of a microtubular fibre as a cell is deformed. Furthermore, many biomacromolecules have been examined to understand the connections between their conformations and their functional roles e.g. the persistence length of DNA is ~0.050 μm, with a moderate dependence on the specific sequence^17^, and it can be used to understand the interaction of DNA with histones. The hope is that the persistence length will also be a useful geometrical tool to characterise ER tubules e.g. to compare their roles in different cells types that have different specialized structures. Some work has previously been performed on the geometry of the ER in cells from the leaves of tobacco plants using confocal microscopy, but here polygons were used to understand the network morphology and the flexibility of the tubules was not characterised^19^.

More recent developments with semi-flexible polymers extended the theoretical models to calculate the impact of hydrodynamics on the transverse displacements ($\langle {\rm{\Delta }}{r}_{\perp }^{2}(t)\rangle$, the transverse mean square displacement as a function of time interval, *t*) of semi-flexible fibres with *no applied tension* $(\langle {\rm{\Delta }}{r}_{\perp }^{2}(t)\rangle  \sim {t}^{3/4})$ and in *tension-dominated* regimes $(\langle {\rm{\Delta }}{r}_{\perp }^{2}(t)\rangle  \sim {t}^{1/2})$ ^14^. Such theories for the fibre dynamics are in good agreement with a range of experimental techniques including fluorescence microscopy, bright field microscopy, photon correlation spectroscopy and microrheology methods^20–22^. More recent theoretical developments consider the active motion of semi-flexible fibres that associate with motor proteins^23–26^. These theories are in a much earlier stage of development than those for the passive fluctuations of fibres, but they predict driven super-diffusive motion of semi-flexible fibres at long time scales ($\langle {\rm{\Delta }}{r}_{\perp }^{2}(t)\rangle  \sim {t}^{\alpha }$, *α* > 1)^23–26^.

In this report, we have used a combination of live cell imaging, conventional diffraction-limited epifluorescence microscopy and stochastic optical reconstruction microscopy (STORM) to investigate the mechanical properties of the ER tubules as well as the dynamic behaviour of individual tubules. We provide evidence supporting the idea of the ER tubules behaving as hollow tubular semi-flexible fibres, with an average outer radius of 44.1 ± 3.2 nm, a persistence length (*L*_*p*_) of 3.03 ± 0.24 μm (4.71 ± 0.14 μm on small length scales between branch points) which corresponds to a very soft membrane bending rigidity (*κ*_*mem*_) of 10.9 ± 1.2 *kT* (17.0 ± 1.3 *kT* on small length scales between branch points) typical of biological lipids in bilayer membranes^27^.

Values for the persistence length of the ER have not been previously reported in the literature and the values we found are intermediate between those of actin filaments (slightly smaller than the actin *L*_*p*_ by a factor of 3–4)^18^ and DNA (much larger by a factor of 60)^16^. Furthermore, the ER persistence length values are one order of magnitude smaller than the size of the cells in which they are investigated. Clearly, the ER is a very soft structure and its contributions to the mechanics of whole cells are expected to be much smaller than those of microtubules, actins or intermediate filaments^28^. Evidence is found for the active modulation of ER dynamics by motor proteins and we expect these active motions will play an important role in the functions of the ER^11^ e.g. the rate of synthesis and subsequent modification of proteins in a *shaken reaction vessel model*.

## Results

### Fibre fitting and the extraction of mechanical properties

**Figure 1:**
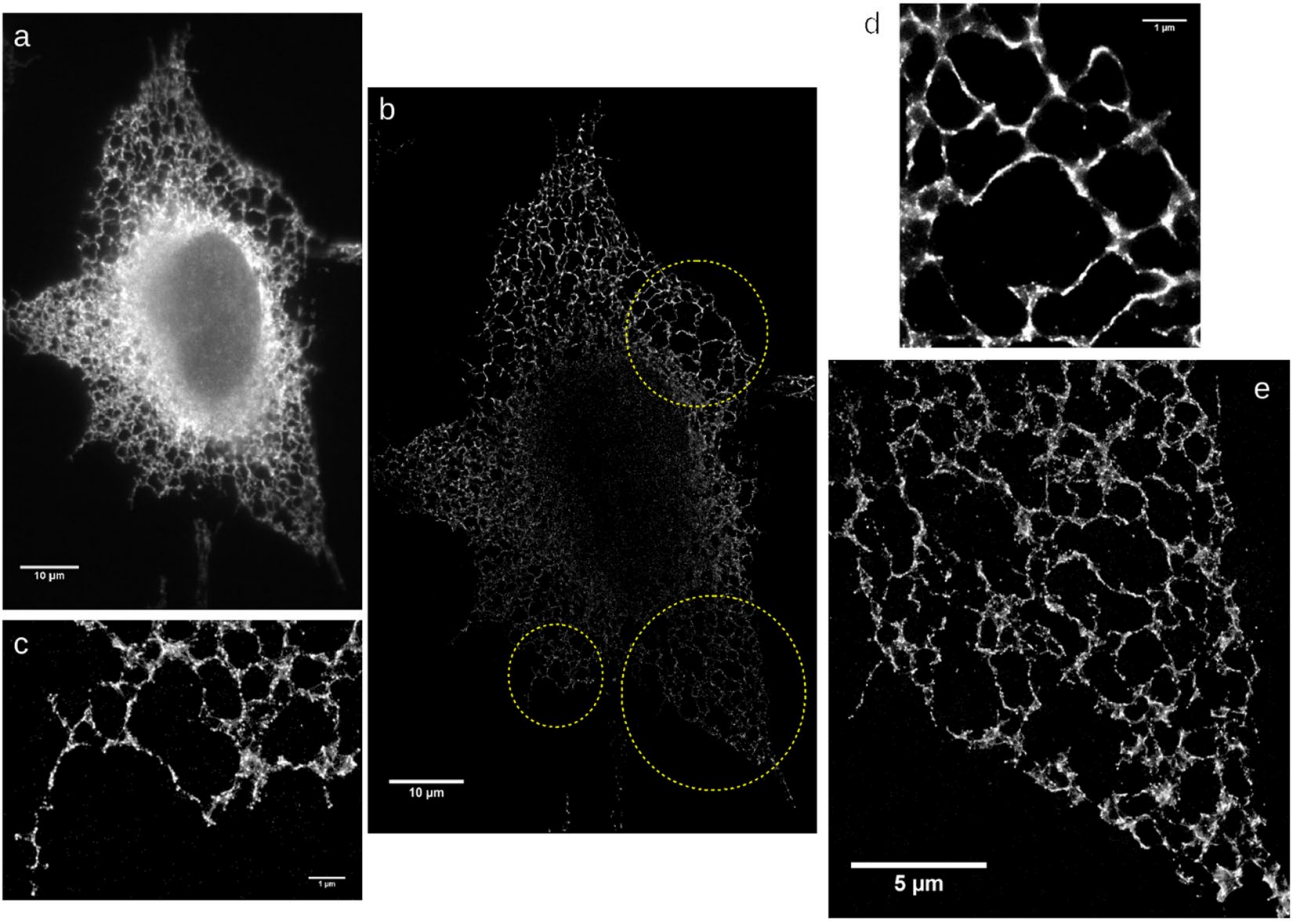
(**a**) Diffraction-limited fluorescent image of an anti-KLC3 stained MRC5 cell and (**b**) the super-resolved STORM image of the same field of view. The gain in resolution is evident from the zoomed field of views (**c**–**e**).

The super-resolved and diffraction limited images of the ER in fixed MRC5 cells (Fig. 1), as well as single frames of transiently transfected live cell videos of MRC5 cells expressing EGFP-ER (Fig. S1), were fitted using FiberApp, an open-source Matlab toolbox that can extract structural information of fibrillar objects in atomic force microscopy and fluorescence images^29^. The contour of the ER tubules was fitted using the embedded procedures that are based on active contour models, which enabled us to calculate the *x-y* position of the tubules, as described in Supplementary Information section 1 (Figs S1 and S2).

**Figure 2:**
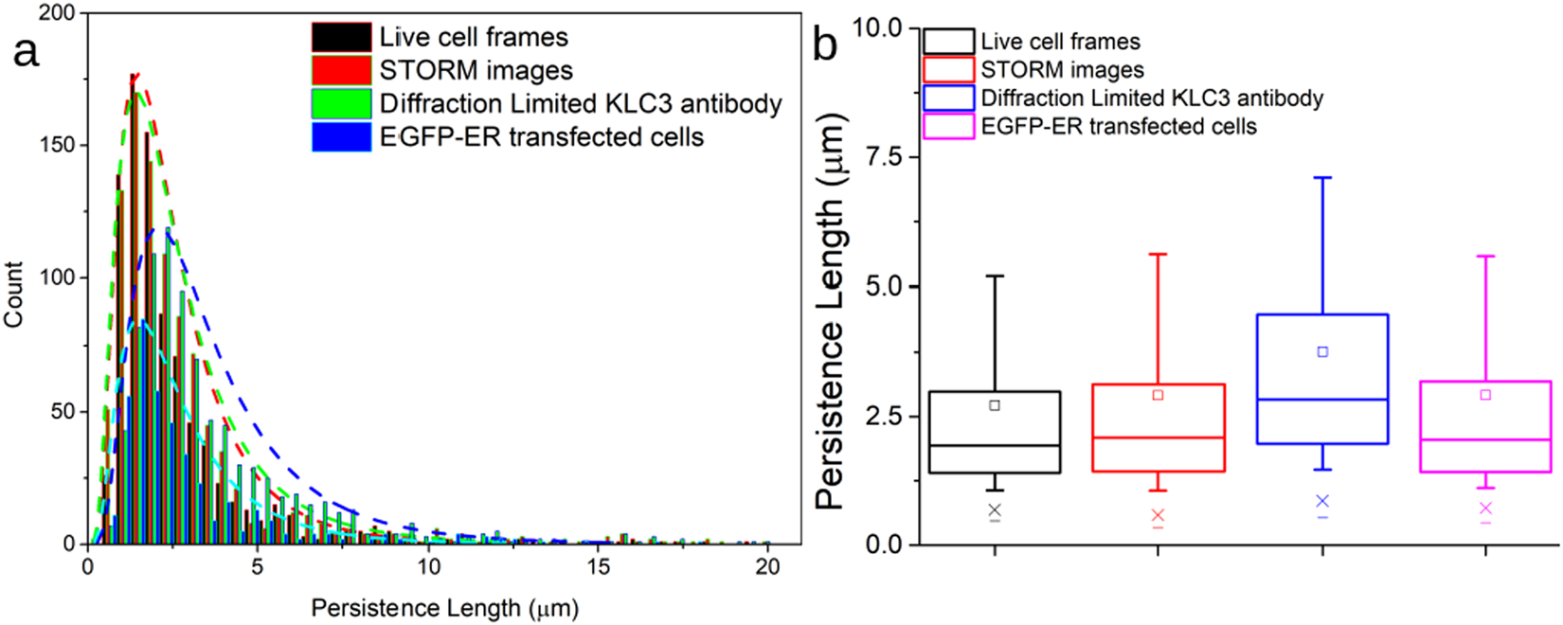
The persistence length (*L*_*p*_) of the endoplasmic reticulum of fixed MRC5 cells (STORM imaging of anti-KLC3, diffraction limited KLC3 antibody and EGFP-ER transfected cells) and live MRC5 cells (conventional diffraction limited fluorescence microscopy). (**a**) A histogram for the distribution of the persistence lengths for the differently labelled cells. The distribution of persistence lengths all follow log-normal distributions for the four differently labelled types of ER images in MRC5 cells. (**b**) The mean persistence length values extracted from the four differently labelled MRC5 cells agree within error (a 95% confidence interval) i.e. diffraction limited live cells, STORM, diffraction limited KLC3 antibody and EGFP-ER transfected fluorescence images.

To compute the persistence length of the ER tubules, the contour length and the end-to-end distance of the active contour extracted from the fluorescent images using FiberApp were used^30,31^ (Fig. S5). The mean square end-to-end distance $(\langle {R}^{2}\rangle )$ can be expressed as

1 $$\langle {R}^{2}\rangle =2{L}_{p}({L}_{c}-{L}_{p}[1-{e}^{-{L}_{c}/{L}_{p}}])$$

where *L*_*p*_ and *L*_*c*_ are the persistence and contour lengths, respectively^32^. The end-to-end distance and the contour length (described in Figure S2) were computed by FiberApp and then the persistence length was extracted by fitting Eq. (1). The mean persistence length found was 3.03 ± 0.24 μm, as shown in Fig. 2. The mean square end-to-end distance has been found to be a more robust measure to calculate persistence lengths from fluorescence microscopy images than direct calculation of angular correlations, because it is less affected by small length scale noise i.e. for a semi-flexible fibre, where *L*_*p*_ ~ *L*_*c*_, most of the information on the angular deflections is contained within the largest wavelength bending modes that also determine <*R* ^*2*^>^31^.

Super-resolved images of fixed MRC5 cells were used in order to measure the outer radius *R* of the ER tubules (Supplementary Information Section 2, Fig. S3). The average outer radius of the tubules was found to be 44.1 ± 3.2 nm.

In addition to the persistence length and the cross-sectional radius, another important property of polymers is their bending rigidity (*κ*_*pol*_), which describes how the applied bending moment (*M*) scales with the curvature ($\frac{{d}^{2}w}{d{r}^{2}}$, where *r* is the deflection and *x* is the distance along the beam) during deformations^28^ i.e. $M={\kappa }_{pol}\frac{{d}^{2}w}{d{r}^{2}}$. For a semi-flexible fibre, *L*_*p*_ can be related to the polymeric bending rigidity using the equipartition theorem for the bending energy,

2 $${L}_{p}=\frac{{\kappa }_{pol}}{{k}_{B}T}$$

where ${k}_{B}T$ is the thermal energy^15,16^. However, it is known that the ER fibres are not uniform solid cylinders, but are hollow with a wall thickness of 3–5 nm^33^. By treating the ER as an elastic hollow cylinder of radius *R* of negligible thickness (the thin hollow cylinder approximation) it is possible to replace equation (2) for the persistence length with an equation based on the anisotropic elasticity of membranes^34^,

3 $${L}_{p}=\frac{2{\kappa }_{mem}\pi R}{{k}_{B}T}$$

where *κ*_*mem*_ is the bending rigidity of the membrane (a two dimensional entity; it is a constant of proportionality between the free energy of the membrane and the mean squared curvature averaged over the membrane), *R* is the radius of the tubule and *k*_*B*_ *T* is the thermal energy. Two advantages of this approach are that it allows the calculation of the bending rigidity of the membranes, which can then be compared with other experiments for their nanomechanics, and that it has been extensively verified with *in vitro* experiments on lipid nanotubules with variable radii^35–38^. Substituting values for the persistence length and the radii of the ER tubules in equation (3) gives a bending rigidity for the ER tubule membranes of 10.9 ± 1.2 *kT* (44.8 ± 4.9 × 10^−21^ J) including the branch points and 17.0 ± 1.3 *kT* (69.9 ± 5.3 × 10^−21^ J) excluding the branch points. Both of these values are comfortably within the range of 3–225 *kT* typically found for lipids in biological membranes^27^ and are thus in good agreement with the expected values.

**Figure 3:**
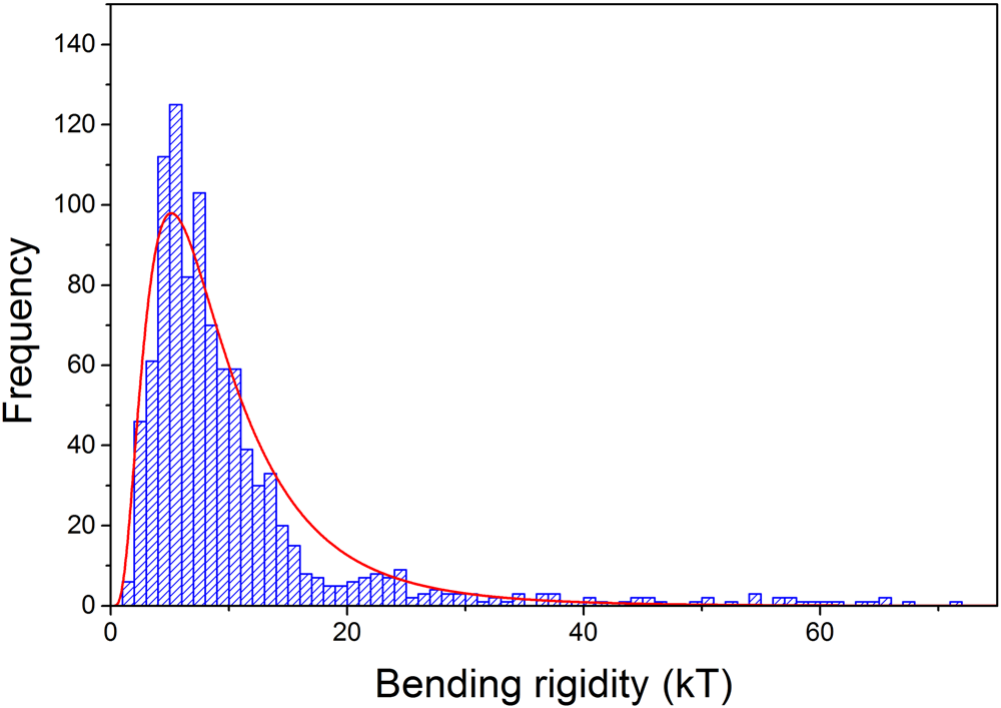
A histogram of the membrane bending rigidities (*κ*_*mem*_) of a tubule in the endoplasmic reticulum calculated using Eq. (3) measured using STORM images of anti-KLC3. A lognormal distribution was fitted to the data. The average membrane bending rigidity of the ER tubules was 10.9 ± 1.2 kT.

The computed membrane bending rigidity (*κ*_*mem*_) from the fitted ER tubules are shown in Fig. 3. The data could be described by a log normal distribution. Such bending rigidities (average 10.9 ± 1.2 *kT*) indicate very soft materials, although they are well within the accepted range for lipid bilayers. For comparison the bending rigidity of bilayer graphene is 1380 kT^39^.

### Tracking of moving ER tubules

**Figure 4:**
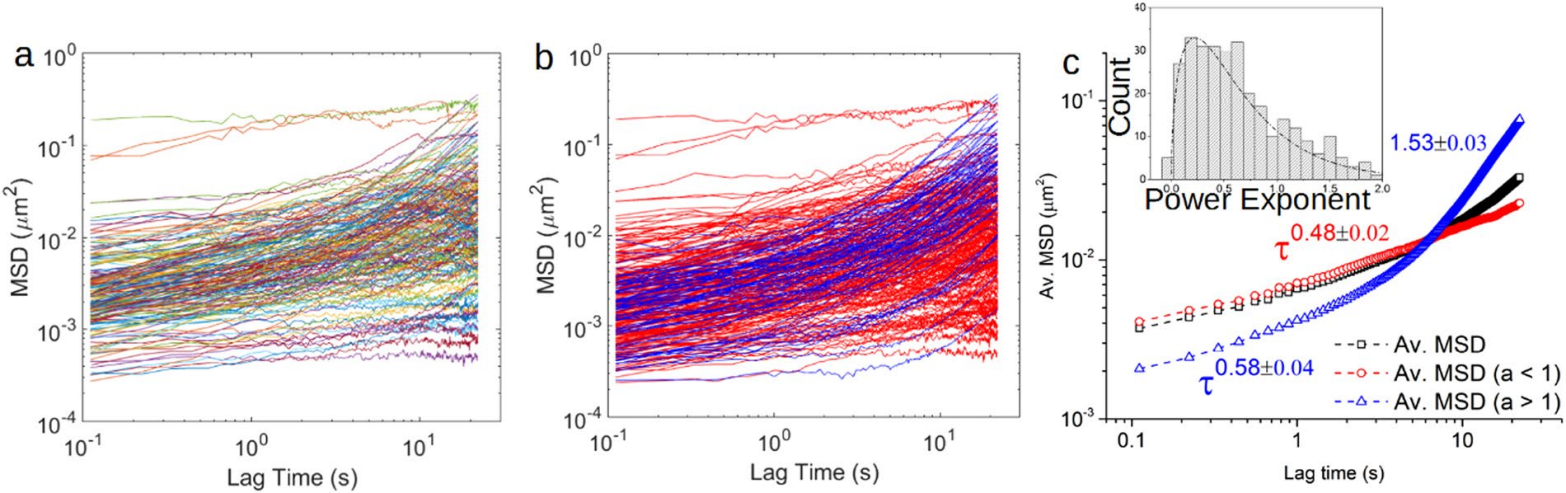
(**a**) The MSD curves as a function of lag time (*τ*) calculated for all the tracked ER tubules. (**b**) There were two distinct populations of MSDs based on their power law exponent, one with *α* < 1 (red) and one where 1 < *α* < 2 (blue) for lag times *τ* > 2 s. The black symbols show the average of the two populations i.e. when the distinction between subdiffusion and super-diffusion is not made. (**c**) The ensemble averaged MSD of the sub-diffusive population could be characterised by a universal power law with *α* = 0.48 ± 0.02, whereas the averaged MSD of the super-diffusive population has a power law of *α* = 0.58 ± 0.04 at small lag times and *α* = 1.53 ± 0.03 at long lag times, τ > 2 s. The inset in (**c**) shows a histogram of the power law exponents extracted from the individual tubules’ MSD curves.

In order for us to further characterise the ER tubular network, live cell videos were used in which the motion of the ER was tracked, as described in Supplementary Information Section 3. Videos where the tracked fibres are tagged using image analysis software are presented in Movies SI1-3. The *x-y* coordinates of the ER tubules along a line drawn perpendicularly to the ER tubule (Fig. S4) were subsequently used to calculate the mean squared displacement (MSD) as a function of lag time for all the tracks. The MSD is defined as

4 $$\langle {\rm{\Delta }}{r}^{2}(\tau )\rangle ={\langle {(x(t+\tau )-x(t))}^{2}+{(y(t+\tau )-y(t))}^{2}\rangle }_{t}$$

where *t* is time and *τ* is lag time. Due to the very small longitudinal movements of the fibres the MSD is a very good approximation to the transverse MSD $(\langle {\rm{\Delta }}{r}_{\perp }^{2}(\tau )\rangle )$ of the fibre’s movements i.e. $\langle {\rm{\Delta }}{r}^{2}(\tau )\rangle =\langle {\rm{\Delta }}{r}_{\perp }^{2}(\tau )\rangle$ $+\langle {\rm{\Delta }}{r}_{||}^{2}(\tau )\rangle \approx \langle {\rm{\Delta }}{r}_{\perp }^{2}(\tau )\rangle$ ^13^. The MSDs obtained from tracking individual MSD tracks are shown in Fig. 4a. In order to characterise the dynamic motion of the ER tubules, a power law of the form $\langle {\rm{\Delta }}{r}^{2}(\tau )\rangle  \sim {\tau }^{\alpha }$ was fitted, which allowed the power exponent *α* of the MSD curves to be calculated. There were two distinct populations of MSDs observed at longer times (>2 s): one *sub-diffusive* population, in which *α* < 1, and another *super-diffusive*, in which 1 < *α* < 2, shown in Fig. 4b. The collective motion of the ER network at longer time scales (>2 s) was responsible for the larger exponents. The two populations were then separated on the basis of their power law exponents at long times and their individually ensemble averaged MSDs are shown in Fig. 4c. A universal power law of *τ* ^0.48±0.02^ characterised the average MSD curve for the sub-diffusive MSD population, whereas a power law of *τ* ^0.58±0.04^ was found for the super-diffusive population in the short lag time regime (up to 2 s) and *τ* ^1.53±0.03^ at longer lag times (above 2 s).

## Discussion

The endoplasmic reticulum is an extremely important organelle in eukaryotic cells, as it is involved in many fundamental cellular processes and its dysfunction has been associated with a number of diseases^40^. However, its structural and mechanical properties are still poorly understood and only with recent advances in super-resolution microscopy and electron microscopy have researchers been able to probe previously unattainable small length scales to reveal new details of the ER’s tubular architecture and dynamics. One example is the recent discovery of the fact that ER sheets in the periphery of the cell are not arranged within sheet-like morphologies, but are densely packed tubules that contain three way junctions, termed ER matrices, and morphological evidence has been provided for the highly dynamic and interchangeable nature of these ER networks^11,12^.

Even though the structure, morphology and distribution of the ER network in a variety of cell lines has been well described, the mechanical properties of the organelle have been little studied. The ER is decorated with a large array of proteins, ribosomes and active motor proteins which could potentially affect its mechanical properties and dynamics^41–43^. We found the persistence length of the ER tubules to be 3.03 ± 0.24 μm, measured from both live and fixed cells. The persistence lengths measured from the four independent experiments (live cell imaging, diffraction limited KLC3 antibody labelling and super-resolution imaging with different labelling protocols) are in good agreement with one another, thus eliminating the possibility of any artefacts due to sample preparation. In addition to the persistence length of the ER tubules, we have also computed their average membrane bending rigidity and it was found to be 10.9 ± 1.2 kT. To check for the possible impact of ER junctions on the persistence length (i.e. a length scale dependent *L*_*p*_ due to chemical heterogeneity, such as junction promoting proteins^3,41^), the analysis was repeated for the stretches of tubule between junction points (Supplementary Information Section 4; Figs S5 and S6). A slight increase in persistence length was observed at 4.71 ± 0.14 μm. The corresponding membrane bending rigidity calculated using equation 3 is also slightly higher at 17.0 ± 1.3 kT, which is expected since the effects of the branching proteins have been removed. The value of the membrane bending rigidity scales linearly with the persistence length (equation 3), and inversely with the radius of the tubule. This provides a clear advantage of super-resolution imaging, since the measured radius of 44.1 ± 3.2 nm is a sub-diffraction length scale for a standard optical microscope, but well above the resolution limit of our STORM microscope (~20 nm).

The persistence lengths and membrane bending rigidities computed from our experiments exhibited a broad distribution indicating the broad range of ER tubule rigidities that are present, which would be consistent with large variations in both the morphology and composition of the ER networks throughout the cell. It has been postulated that the curvature of the tubules is dictated by the interplay of two sets of proteins, R-type and S-type proteins. R-type proteins, such as reticulons and DP1/Yop1p, promote tubule formation, whereas S-type proteins, such as atlastins, Sey1p and Climp63, promote junctions between tubules and what were known as sheets^3,41^. However, the complete set of proteins which determine the ER’s morphology is still unknown. Additionally, the ER interacts physically with a number of organelles, such as endosomes, and the ER’s motion has been directly linked to the microtubular cytoskeleton of the cell^6^, with both types of interactions contributing to the overall morphology of the network^43^. It is therefore concluded that the distribution of persistence lengths and membrane bending rigidities observed can be attributed to an intricate interplay between the aforementioned factors which all perturb the extremely soft tubular structures that the ER forms.

In addition to the mechanical properties of the ER network, its dynamic behaviour was also investigated using live cell videos and tracking software. Two distinct mean square displacement (MSD) populations were identified as a function of lag time (*τ*) at longer lag times (*τ* > 2 s): one that exhibits *sub-diffusive* motion (0 < *α* < 1) and one that exhibits *super-diffusive sub-ballistic* motion (1 < *α* < 2). The sub-population of super-diffusive sub-ballistic tracked ER tubules, could potentially be affected by the collective motion of the ER network as a result of the cell’s motion or, more likely, could be the direct result of an active process driven by motor proteins or the translocation of the tubule as a result of a transient interaction with microtubules or vesicles^11^. By filtering out tracks with super-diffusive motions in their MSDs at long times (>2 s), we observed a universal power law with an exponent *α* of 0.48 ± 0.02 for all the other tracks, which agrees within error with a theoretical prediction for semi-flexible fibres under tension, anchored on each end, equation (5)^14^. Short lived tracks (<5 s) were not considered in the analysis. Our frame rate was too slow to allow tracking of the fastest moving tubules, and so these were also not included in the datasets. These tubules were possibly in contact with other components of the cell that were undergoing rapid translocation. We thus expect there is a third population of extremely fast tracks that have super-diffusive sub-ballistic motions. Whether this third population is distinct from the other population of super-diffusive sub-ballistic tracks we have already measured would require additional research.

**Figure 5:**
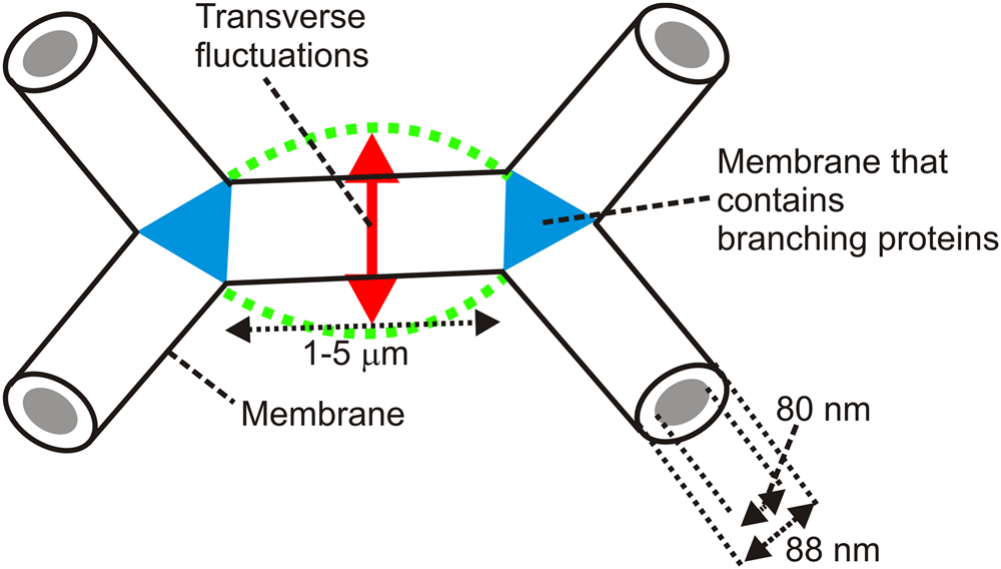
A schematic diagram that shows a fragment of the ER network’s tubular structure (not to scale). The distance between the branch points is in the range 1–5 μm, the inner tubule diameter is 80 nm and the outer tubule diameter is 88 nm. Transverse fluctuations were measured based on the transverse mean square displacements $(\langle {\rm{\Delta }}{r}_{\perp }^{2}(\tau )\rangle )$, shown in Fig. 4. The branched regions are labelled in blue in which specific S-type proteins are thought to occur^41^.

A schematic diagram of a part of the ER tubular network experiencing transverse fluctuations is shown in Fig. 5. A specific prediction for the transverse mean square displacements $(\langle {\rm{\Delta }}{r}_{\perp }^{2}(\tau )\rangle )$ of a fibre under tension is given by

5 $$\langle {\rm{\Delta }}{r}_{\perp }^{2}(\tau )\rangle ={(\frac{{k}_{B}T}{\sigma })}^{3/4}\frac{{L}_{p}^{1/4}{\tau }^{1/2}}{2{\eta }^{1/2}}$$

where *τ* is the lag time, *σ* is the stress in the fibre and *η* is viscosity of the solvent^14^. Fitting the gradient of the MSD which has a *τ* ^*1/2*^ scaling in Fig. 4c gives the average stress (*σ*) in the ER tubules as 58.3 ± 9.1 Pa. This value seems reasonable, since it corresponds to an average tension force of around 10 fN applied longitudinally to each ER tubule and is well within the range that can be exerted by single motor proteins, fN-pN. It does imply a slight correction to the persistence lengths calculated using equation (1). We estimated this using an analytic expression for the non-linear elasticity of a semi-flexible filament^13^, which gave an effective strain of 0.03 for the tubules under tension. This strain in turn implies a ~3% correction to the persistence length (it is overestimated by this amount) given by equation (1), which is within the experimental errors and can be neglected. The tension force is three orders of magnitude smaller than that needed to form tethers (i.e. large morphological changes) in reconstituted ER in *in vitro* experiments with optical tweezers (18.6 ± 2.8 pN)^44^. A characteristic length scale is associated with the tension stress given by ${l}_{t} \sim \sqrt{\frac{{\kappa }_{pol}}{\sigma }}$, due to the competition between bending and tension, and it is equal to (3.7 ± 0.6)×10^−14^ m for the tubules^13^. At length scales above *l*_*t*_ the dynamics are expected to be dominated by tension and thus the ER tubules are expected to be described by equation (5), since the motion of micron sized fibres are well above this limit. Furthermore, the aforementioned stresses are well above the $\sigma \gg {k}_{B}T{L}_{p}/{L}^{2}$ limit for the validity of equation (5) for the semi-flexible dynamics under a passive tension^13,14^ (*L* is the contour length of the fibre). The time dependence in equation (5) looks Rouse-like (i.e.$\langle {\rm{\Delta }}{r}^{2}(\tau )\rangle  \sim {\tau }^{1/2}$, a scaling result for flexible polymeric chains with no tension^45^), but it is valid for semi-flexible polymers (the image analysis provides clear evidence that the ER tubules are semi-flexible, since their persistence length is on the order of the contour length) and the prefactors in the equation have a different scaling compared with the flexible polymer result^14^. More subtle variations of the persistence length of semi-flexible fibres are known to occur under tension^46^, but it is expected that equation (3) is a good first approximation for the membrane bending rigidity (κ_mem_) of the ER tubules and the persistence lengths calculated (due to the geometrical definition of the persistence length and the use of direct image analysis) are independent of tension effects. No significant population of ER tubules was found that was not under tension i.e. a population that neither agreed with the predictions of equation (5) nor had super-diffusive motions driven by motor proteins.

Based on the analogy with a hexagonal honeycomb foam, a first approximation can be made for the Young’s modulus and shear modulus of the full ER network architecture^47^. The in-plane Young’s modulus (*E*_*in*_) of the ER network (i.e. in the plane of the 2D honeycomb) scales as

6 $${E}_{in} \sim {(\frac{2r}{L})}^{3}E$$

where *2r* is the tubule thickness, *E* is the modulus of the tubules and *L* is the distance between branches. This predicts that the in-plane Young’s modulus of the ER network is a million times weaker than that of a single tubule i.e. *E*_*in*_ ~ 10^*−*6^ *E*. The in-plane shear modulus is also expected to be extremely soft *G*_*in*_ *~* *0*.*25* × 10^*−*6^ *E*. The out-of-plane Young’s modulus has a (*2r/L*)^1^ scaling, so *E*_*out*_ ~ 10^*−*2^ *E* and the out-of-plane shear moduli (*G*_*out*_) are also of a similar order of magnitude. In conclusion, the ER network has an extremely soft structure compared with other cytoskeletal materials (e.g. actin, intermediate filaments and microtubules) and the in-plane deformations (in the plane of the ER network, coincident with the plane of the cell in the current experiment) are expected to be more pronounced that the out-of-plane deformations (out of the plane of the ER network). More sophisticated models are possible to describe the non-linear mechanical properties of the ER tubules using the analogy to a foam structure, with more accurate local geometrical properties for their cross-linked networks (e.g. bond angles, coordination numbers, tubule thicknesses etc.)^13^. In *tension*, non-linear elasticity is expected for the ER tubules at large deformations, whereas in *compression* linear elasticity is expected to be followed by tubule buckling and compaction at large deformations^47^ (a collapse of the network structure with a substantial loss in volume). Nevertheless, the ER structures are so soft that they will provide only a very small contribution to the overall mechanics of a single cell when compared with the contribution of cytoskeletal filaments, e.g. actins and microtubules, which will bear the majority of any imposed stresses^27^.

In addition, how motor proteins give rise to both passive tension and the active motions of biological fibres has started to be modelled^23,25^, but more developments are needed to make these studies directly relevant to ER tubules. Here we have provided a simple robust experimental determination of the mechanical properties of single tubules and we would hope to elaborate on these measurements in future studies. We expect that the highly soft dynamic network of the ER tubules plays an important role in the biological functions of the ER e.g. it could lead to an efficient mechanism of convective transport for mixing of reactants during biomolecule synthesis (such as the synthesis of proteins by ribosomes associated with the tubular membranes, and the processing of proteins within the ER tubule lumen) i.e. a *shaken reaction vessel model*.

The structure, mechanical properties and dynamic behaviour of the ER are important in order to fully understand its functions. Further research into identifying the complete set of proteins that dictate the morphology of the tubular network and define the roles of the ER is needed^4^. Furthermore, the ER morphology and its functions can vary between cell types, so it will be important to explore its properties in a wider range of cells e.g. the sarcoplasmic reticulum in cardiac cells.

## Conclusions

The ER forms a remarkably intricate and complex network of interconnected tubules in eukaryotic cells that stretches from the nuclear envelope to the cell periphery. We used a combination of diffraction-limited fluorescence imaging of fixed and live cells, as well as super-resolution STORM imaging of fixed cells, to calculate the mechanical properties of the ER and probe the dynamic behaviour of the tubules. We found the radius of the ER tubules was 44.1 ± 3.2 nm, the average persistence length was 3.03 ± 0.24 μm (4.71 ± 0.14 μm between branch points) and the average membrane bending rigidity was 10.9 ± 1.2 kT (17.0 ± 1.3 kT excluding branch points). Furthermore, in conjunction with the MSD analysis, we have concluded that there is a population of active ER tubules whose motility is driven either directly or indirectly by motor proteins in the cytoplasm and a larger population of ER tubules that only experience a passive tension. These motions may impact the function of the ER e.g. the rate of protein synthesis in a *shaken reaction vessel model*. The transverse fluctuations of the tubules under tension are in good agreement with a theory for the hydrodynamics of semi-flexible fibres. No population of ER tubules was found that was not under tension.

## Materials and Methods

### MRC5 cell culture and preparation for fixed cell analysis

MRC5 foetal lung fibroblast-like cells were used for STORM and diffraction limited fluorescence imaging experiments. Cells were cultured in T75 flasks at 37 °C with 5% CO_2_. The cell cultures were split at ~80% confluency using trypsin/EDTA and were cultured using Eagle’s Minimum Essential Medium (EMEM), complemented with 2 mM L-Glutamine, 1% Non-Essential Amino Acids (NEAA), 10% Foetal Bovine Serum (FBS), streptomycin and penicillin. Cells intended for diffraction limited imaging were cultured on No 1.5 coverslips for 48 hrs prior to fixing, whereas cells intended for STORM imaging were cultured for 48 hrs prior to imaging in Mattek glass bottom dishes (uncoated 35 mm dish, No. 1.5 coverslip, 10 mm glass diameter).

When the desired confluency was achieved, cells were quickly rinsed with PBS and fixed using a 3% Formaldehyde/0.05% Glutaraldehyde solution in PBS for 15 min at room temperature. The reaction was quenched using a freshly made sodium borohydride solution (1 mg/ml in PBS: 3 washes, 5 min each at room temperature). The cell membrane was then permeabilised by incubating with a 0.5% (v/v) Triton X100 solution in PBS for 1 hour at room temperature, followed by three 5 min PBS washes. Non-specific binding of antibodies was minimised by incubating with a 4% BSA, 0.1% Triton X100 solution in PBS blocking buffer for 15 min at room temperature. The endoplasmic reticulum was stained by using a rabbit monoclonal antibody to kinesin light chain 3 (KLC3) (Abcam ab180523) at a 1:400 dilution of the stock solution in blocking buffer. This antibody strongly labels the ER, even though it is likely recognising a protein that is not KLC3 (data not shown). Cells were incubated for 20 min at room temperature with the primary antibody and subsequently washed 3 times with PBS (5 min each) to remove free antibodies. A 15 min incubation at room temperature with a 1:800 dilution of F(ab’)2 fragments, donkey anti-rabbit Alexa Fluor 647 (Abcam ab181347) secondary antibody solution in blocking buffer was used to fluorescently label the primary antibodies. Free fluorophores were removed by washing 3 times with PBS (5 min). Coverslips intended for diffraction limited studies were mounted on microscope slides using Prolong Gold (Invitrogen) and left overnight to set, whereas glass bottom dishes intended for STORM imaging were transferred directly to the super-resolution laboratory for imaging.

MRC5 cells were also transiently transfected using an ER-targeted EGFP construct^6^. Briefly, cells were cultured on No 1.5 coverslips in a 12 well plate, using antibiotic free media until ~50% confluency was reached. FUGENE was added to Optimem media, which was gently mixed for 3 min prior to the addition of DNA. A concentration of 0.5 μg/ml per coverslip was used for the DNA construct and a ratio of 1:3 with relation to FUGENE was maintained. The DNA-FUGENE solution in Optimem was incubated for 20 min at room temperature and added dropwise into the wells. Cells were further cultured for 24 hours to reach sufficient transfection efficiency. Transfected cells were fixed and mounted on microscope slides as described above using 3%PFA/0.05% Glutaraldehyde.

### Live cell imaging

To image the ER in living cells, MRC5 cells were grown on Mattek 35 mm dishes and transiently transfected with EGFP-ER^6^ 18 hours before imaging. A mixture of 70 ng EGFP-ER, 140 ng mCherry Rab5 and 2.59 µg of pBluescript (as carrier of DNA^48^) were combined with 8 µl FUGENE in 280 µl OptiMEM and added to each 35 mm dish. The following day, the cells were incubated in 200 nM SiR-tubulin^49^ (Spirochrome; Stein-am-Rhein, Switzerland)) diluted in fresh EMEM/FBS media (see above) for an hour at 37 °C, 5% CO_2_. Cells were then imaged using a DeltaVision OMX microscope (GE Healthcare) in wide-field mode, at 37 °C and 5% CO_2_. All channels were acquired simultaneously using three independent Evolve liquid cooled EMCCD cameras (Photometrics), but only the ER images were used here. EGFP was imaged with a 488 nm band of excitiation light from a solid state illuminator together with the BGR-FR filter drawer containing a quad-band dichroic cascade and 528/48 nm emission filter. An Olympus Plan Apochromat 100x/1.4 PSF oil immersion objective lens was used (1.514 refractive index immersion oil). A sequence of 400, 16 bit, 512 × 512 pixel images were collected at a frame interval of 115.15 ms, at 12.983 pixels/µm. Image sequences after acquisition were processed in Fiji.

### Super-resolution fluorescence imaging

Super-resolution STORM images were acquired using our custom built setup, which has undergone some minor modifications from what was described in our previous work^31,50^. Specifically, the four laser beams were delivered through an oscillating multimode optical fibre, instead of a free space configuration, using a dedicated speckle scrambler. Briefly, our setup comprises of four laser lines (405, 488, 561 and 647 nm), which were fibre coupled into a multimode optic fibre (Thorlabs M45L02) using a combination of dichroic and dielectric mirrors. A vibrating motor was attached to the optical fibre, which was used to homogenise the beam and ensure homogeneous illumination of the fluorescent sample. The use of an optical fibre ensured a concentric and more consistent intensity distribution of the different wavelengths, which is important in multi-colour super-resolution fluorescence imaging experiments. The biological samples were illuminated using an epillumination geometry through an Olympus 100× TIRF oil immersion objective (Olympus UAPON 100XOTIRF, NA 1.49) and the fluorescence signal was recorded on a Hamamatsu ORCA-Flash4.0 v2 sCMOS camera (bin = 2, exposure = 10ms)^51^. Binning the pixels of the sensor into a 2 × 2 configuration in combination with the 100× TIRF objective lens resulted in an effective pixel size of 130 nm. In order for the Nyquist criterion to be satisfied, the pixel size of the camera sensor should ideally be 2.3 times smaller than the diffraction limit of the equipment. In this case, since a far red fluorophore was used (Alexa Fluor 647) with an emission peak at 665 nm, the diffraction limit was ~300 nm. Hence, binning the sensor in a 2 × 2 configuration ensured the effective pixel size was as close as possible to the ideal pixel size to satisfy the Nyquist criterion^52^.

MRC5 cells cultured for STORM imaging were grown in Mattek glass bottom dishes for 48 hrs prior to fixing to reach the desired confluency. The imaging buffer used to accommodate efficient photo-switching of the embedded fluorophores consisted of 10 mM monoethanolamine (Cysteamine, 30070 Sigma-Aldrich), 10% (w/v) glucose (Sigma Aldrich), 0.5 mg/ml glucose oxidase (G2133 Sigma-Aldrich), 40 μg/ml catalase (C40 Sigma-Aldrich) in a 50 mM Tris, 10 mM NaCl buffer at pH 8.0 (adjusted using NaOH)^53^. A total of 50,000 frames were collected at 100 fps on a ~50 × 50 μm wide field of view, which ensured adequate reconstruction of super-resolved images of single whole cells. The diffraction limited data were subsequently analysed using ThunderSTORM, an ImageJ plugin developed for STORM microscopy^54^. Lateral thermal drift was corrected using ThunderSTORM. Poor localisations, based on their positional uncertainty and fitted sigma values, were filtered out in post-processing. The super-resolved images were reconstructed using the Averaged Shifted Histograms function in ThunderSTORM using a pixel size of 10 nm, as shown in Fig. 1.

## Electronic supplementary material

Supplementary information
