# Supplementary material for: The flexibility and dynamics of the tubules in the endoplasmic reticulum
Source: Sci Rep. 2017 Nov 28;7:16474. doi: 10.1038/s41598-017-16570-4 (PMC5705721; doi:10.1038/s41598-017-16570-4)
Supplement: Supplementary file 1 — Supplementary information [file 41598_2017_16570_MOESM1_ESM.pdf]

Supplementary Information

The flexibility and dynamics of the tubules in the endoplasmic reticulum

Pantelis Georgiades[1,2,3], Victoria J. Allan[2,3*], Graham D. Wright[4], Philip G. Woodman[2], Parinya Udommai[1], Manloeng A. Chung[1], Thomas A. Waigh[1,3⊥]

[1]Biological Physics, School of Physics and Astronomy, The University of Manchester, Manchester, UK
[2]Faculty of Biology, Medicine and Health, Manchester Academic Health Science Centre, The University of Manchester, Manchester, UK
[3]Photon Science Institute, The University of Manchester, Manchester, UK
[4]IMB Microscopy Unit, Institute of Medical Biology, A*STAR, 8A Biomedical Grove, #06-06 Immunos, Singapore 138648

Section 1

ER Tubule tracking with FiberApp: Prior to loading the images into FiberApp a 1.4 pixel Gaussian kernel was applied to them, in order to reduce small brightness variations along the fibres' contours, which would otherwise result in a local artificial increases in curvature. Furthermore, for diffraction limited images, the background was removed using ImageJ's built in function, using a 5 pixel rolling ball radius. For STORM images, no background subtraction was performed as STORM images have inherently zero background, since only blinking fluorophores are localized. This ensured maximum contrast between the fluorescent tubules and the background, which enabled FibreApp to be more effective in tracking the contour of the fibres, as seen in Fig. S1. Additionally, an example of a window used for fits is shown in Fig S2. Note that at contour lengths much bigger than the persistence length (>>3 μm), the persistence length calculated from equation (1) does not depend on the exact size of the contour length chosen. Thus we are fairly unconstrained in our choice of contours in figure S2, as long as they are sufficiently long and there are a reasonably large number of contours chosen, we expect that the average persistence length is accurately calculated.

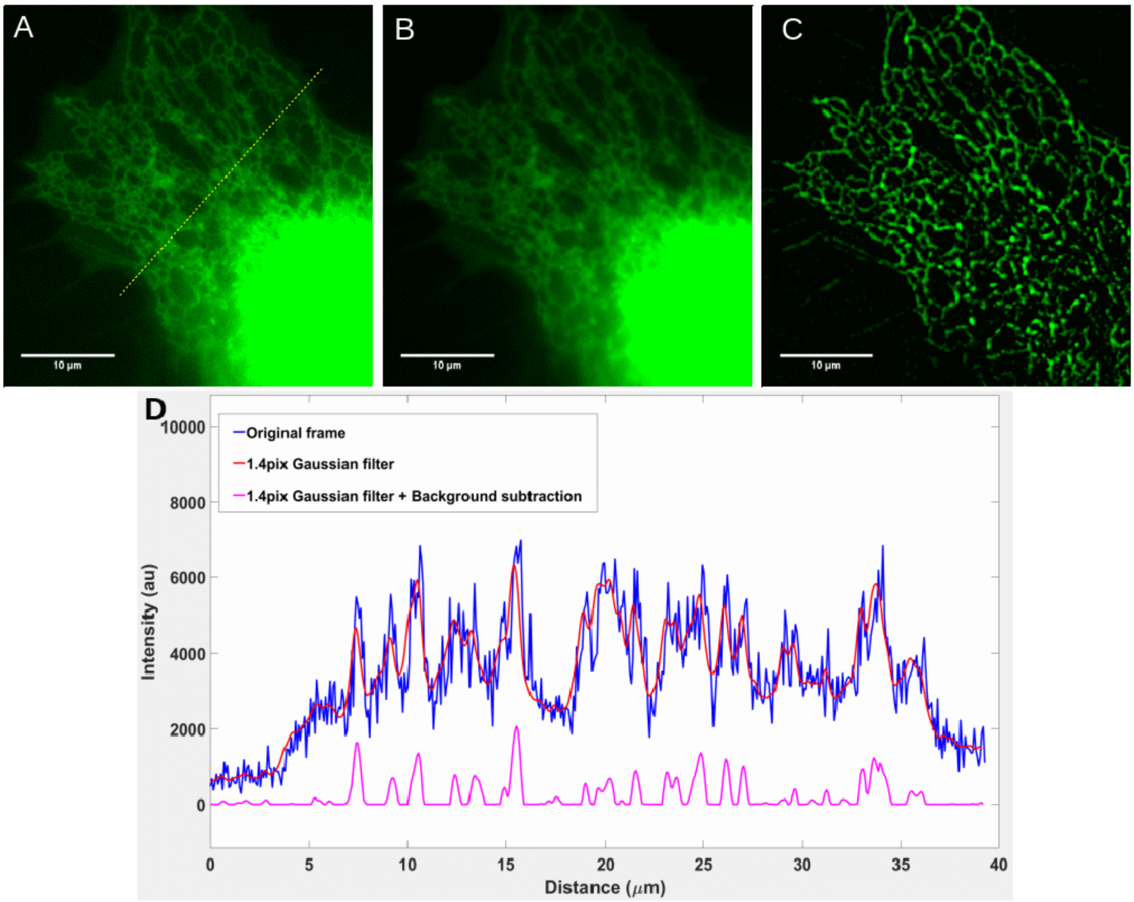

Figure S1. An MRC5 cell was transiently transfected with EGFP-ER and imaged using conventional fluorescence microscopy. A 1.4 pixel Gaussian kernel (B) was applied to the original frame (A) and then, the background was subtracted (C), using a 5 pixel rolling ball radius. The intensity profile along the line shown in (A) between the three images is compared in (D) (yellow dotted line in A). The contour fitting algorithm performs significantly better with minimal background, so the aforementioned procedure was applied to all the diffraction limited images, which were then fitted with FiberApp (1). STORM images have inherently zero background, so background subtraction was not applied.

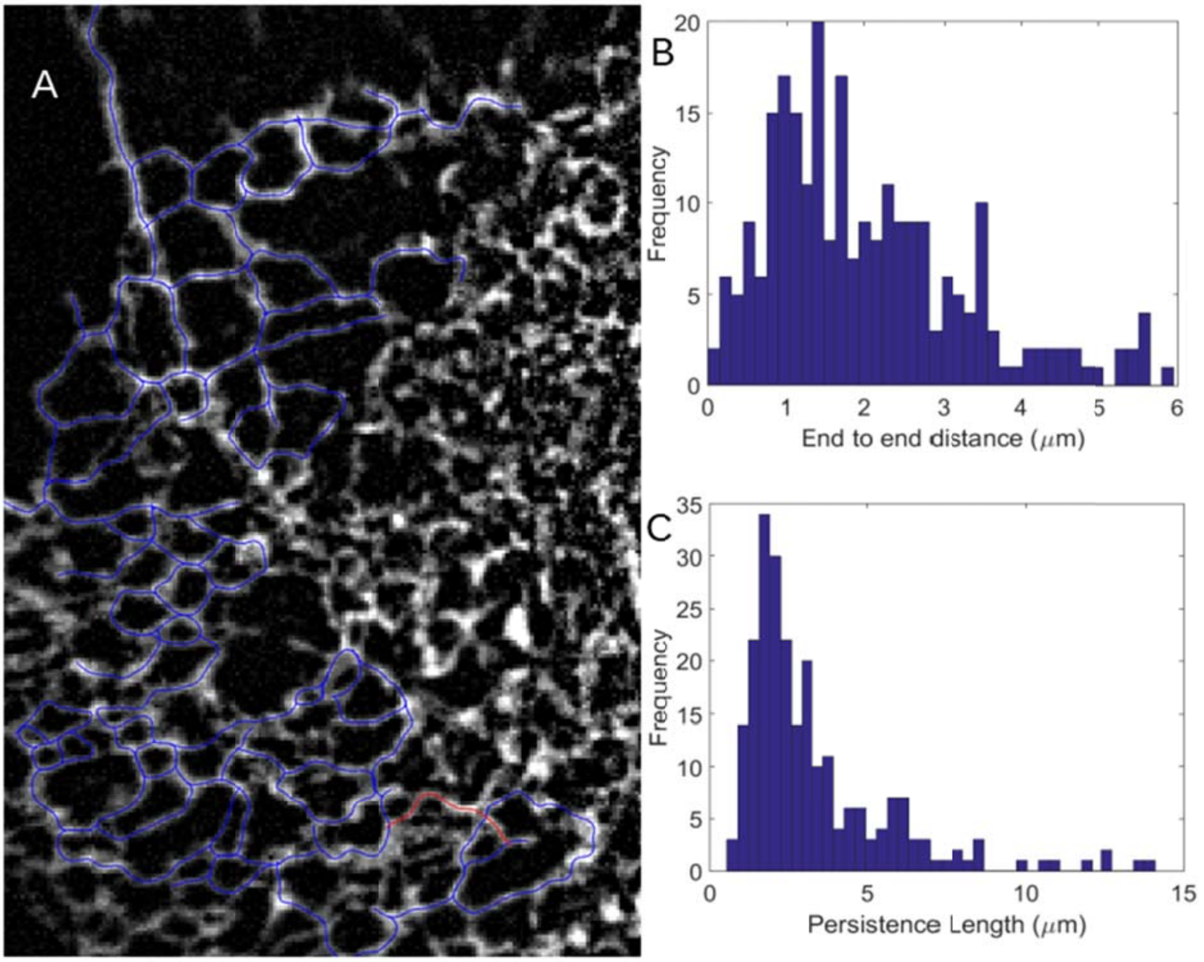

Figure S2. A) The diffraction limited image shows a fixed MRC5 cell labelled with anti-KLC3 and imaged using an epifluorescence microscope. An example of the fibre fitting window of FiberApp (A) with a number of fitted contours. Blues lines show the network of all ER tubules sufficiently well resolved to be used in the analysis. The red line shows a single contour used for the analysis. The end-to-end distance (B) (R_N, figure S5) and the persistence length (C) distributions extracted from this image are shown on the right. Only sufficiently well resolved tubules were fitted.

Section 2

Measurement of the ER tubules radius

Super-resolved STORM images of the ER tubular network were used to calculate the radius of the tubules, as shown in Fig S3. The perpendicular intensity profile of well discerned tubules was fitted with a Gaussian function and the full width at half maximum (FWHM) of the distribution was recorded for a total of 45 ER tubules from 6 cells. The weighted average diameter was found to be 88.1±3.2 nm.

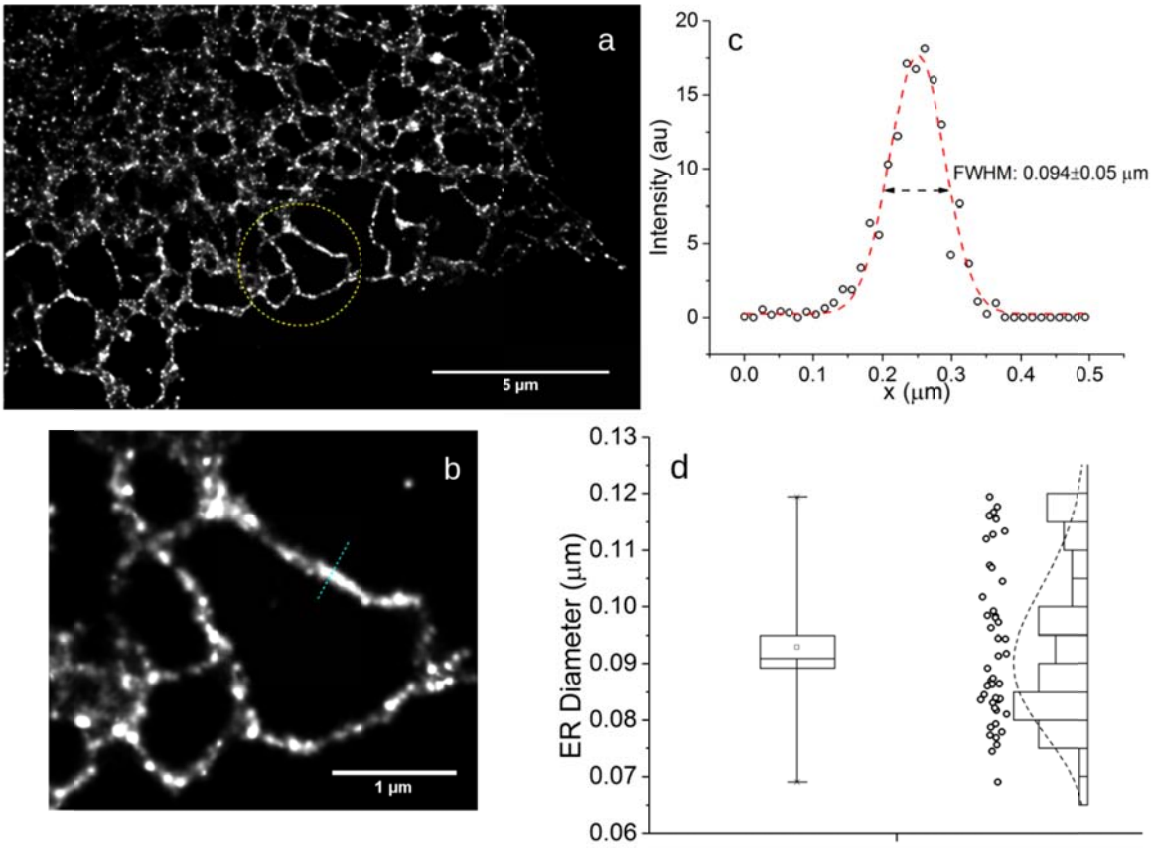

Figure S3. (a) STORM images of the ER were used to calculate the diameter of the tubules. (b) Well discerned ER tubules were used and their perpendicular intensity profiles were fitted with a Gaussian function and (c) their full width at half maximum (FWHM) was recorded (the x axis corresponds to the blue dotted line on b). (d) The distribution of diameters calculated using fits such as that shown in (c). The average diameter from the FWHM of a total of 45 tubules was found to be 88.1±3.2 nm.

Section 3

Tracking of the ER in live cell videos: Transiently transfected MRC5 cells were used in live cell experiments using conventional fluorescence microscopy, in which the dynamic behaviour of the ER was recorded. A total of 16 cells were used. In order to extract the motion of the ER, the following procedure was followed:

- A 1.4 pixel Gaussian filter was applied to all the frames of the video.
- The background was subtracted using a 5 pixel rolling ball average using ImageJ's built in function.
- Images were loaded into Matlab. A custom written algorithm looped through the frames and extracted the intensity profile between two user defined points on the image, chosen to be perpendicular to ER tubules in this case.
- A Gaussian function was fitted in all the intensity profiles, in order for the centre of the distribution to be determined, as shown in Fig S4. This corresponds to the apex of the contour of the tubules.
- The x-y coordinates of the tubules were extracted and subsequently used in MSD calculations.

Additionally, example videos of tracked ER tubules are available in Movies S1, 2 and 3 .

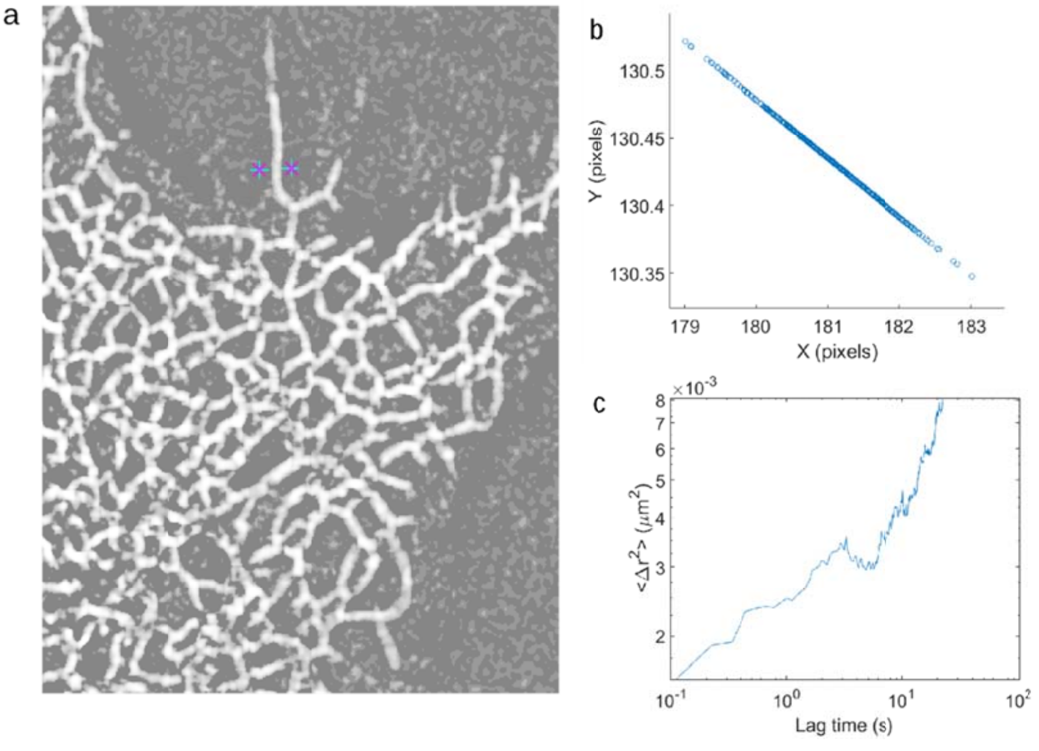

Figure S4. Workflow of ER tracking. (a) The first frame of the video sequence is displayed and two points are chosen by the user shown as blue crosses. (b) The algorithm then loops through all the frames in the video,

extracting the centroid of the intensity distribution by fitting a gaussian function along the line between the two points, which corresponds to the apex of the tubule's contour, extracting the x-y coordinates of the ER. (c) The tracks of 314 tubules were subsequently used to calculate their mean square displacements ($\langle\langle \Delta r^2(t) \rangle\rangle$) as a function of lag time.

Section 4

Length scale dependence of the ER tubule persistence length

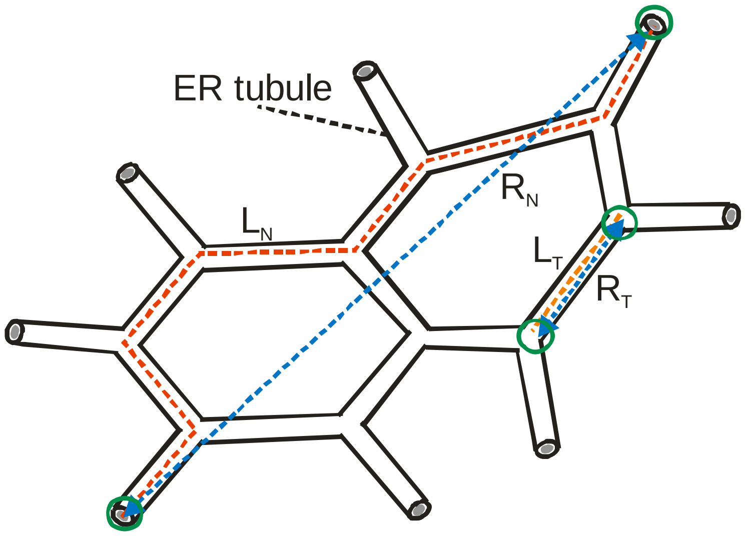

Figure S5. A schematic diagram of a fragment of the tubular ER network. L_N and L_T are the contour length between two points on the network and the contour length between two branch points respectively. R_N and R_T are the end-to-end distances between two points (green circles) on the network and the end-to-end distance between two branch points respectively. Thus using eq. (1), the pairs of (L_N, R_N) and (L_T, R_T) allow the calculation of the persistence length at larger length scales (including the effect of the branching points) and that at shorter length scales (excluding the effects of branching proteins) respectively.

Values for the ER network persistence length were calculated using equation (1) based on pairs of values of L_N (the contour length between two points) and R_N (the end-to-end distance between two points), figure S5. Comparison was also made for the ER tubule persistence length calculated between branch points (using pairs of values L_T and R_T, for the contour length between branch points and the end-to-end distance between branch points respectively) and the results are shown in figure S6.

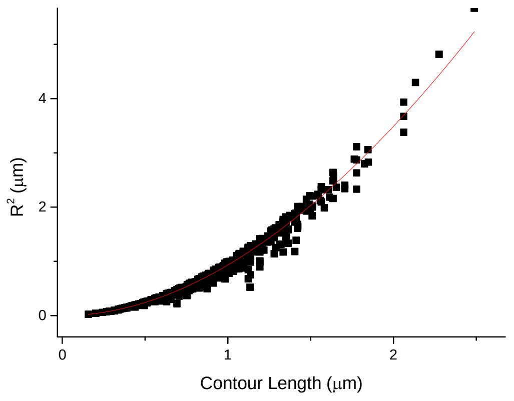

Figure S6. Square end-to-end distance (R^2) of the ER tubules between branch points as a function of their contour length (L) i.e. measurements of R_T^2 and L_T from figure S5. 950 tubules from 8 separate cells were analysed. A fit of eq. (1) is shown in red and it provides a value for the persistence length of the ER tubules between the branch points of 4.71±0.14 μm.

Movies

Three examples of movies (S1, S2 and S3) are provided. They are from transiently transfected MRC5 cells and were imaged using conventional fluorescence microscopy as described in the main text. The blue circles indicate the points at which the mean square displacements of the tubules were measured as described in Section 3.

References

1. Usov, I., and R. Mezzenga. 2015. FiberApp: An open-source software for tracking and analyzing polymers, filaments, biomacromolecules, and fibrous objects. Macromolecules. 48: 1269–1280.
